# The Anticancer Effect of a Flavonoid-Rich Extract of Bergamot Juice in THP-1 Cells Engages the SIRT2/AKT/p53 Pathway

**DOI:** 10.3390/pharmaceutics14102168

**Published:** 2022-10-11

**Authors:** Alessandro Maugeri, Caterina Russo, Laura Musumeci, Giovanni Enrico Lombardo, Giovambattista De Sarro, Davide Barreca, Santa Cirmi, Michele Navarra

**Affiliations:** 1 Department of Health Sciences, University “Magna Græcia” of Catanzaro, 88100 Catanzaro, Italy; 2 Department of Chemical, Biological, Pharmaceutical and Environmental Sciences, University of Messina, 98166 Messina, Italy; 3 Fondazione “Prof. Antonio Imbesi”, 98123 Messina, Italy

**Keywords:** *Citrus*, bergamot, flavonoids, natural products, *Citrus bergamia*, cancer, acute myeloid leukemia, sirtuins, SIRT2, apoptosis

## Abstract

Novel targets are constantly sought to fight hematologic malignancies. In this regard, high levels of SIRT2 expression are associated with unfavorable prognosis of acute myeloid leukemia. The interest in the plant kingdom has allowed the identification of ever-new anti-leukemic agents. *Citrus × bergamia* (bergamot) was proved to possess anticancer properties, yet no evidence is available regarding leukemia. For the first time, we studied the potential anti-leukemic effect of a flavonoid-rich extract of bergamot juice (BJe) in THP-1 cells, investigating the underlying mechanisms. Our findings showed that BJe reduced THP-1 cell proliferation, without affecting that of primary PBMCs, blocking the cell cycle in S phase and inducing apoptosis. Triggering of both extrinsic and intrinsic apoptotic pathways was witnessed by cleavage of caspase-8 and -9, which in turn activated caspase-3 and PARP. Interestingly, the increased p53 acetylation in THP-1 cells underlies SIRT2 inhibition by BJe, that was proved also in the isolated enzyme. Moreover, BJe hampered SIRT2 also by lowering its gene expression. Finally, BJe reduced AKT phosphorylation, which we hypothesized being the joining link between SIRT2 and p53, that play a pivotal role in BJe-induced cell cycle arrest and apoptosis in THP-1 cells. Our results suggest BJe as a potential anti-leukemic agent, via targeting of the SIRT2/AKT/p53 pathway.

## 1. Introduction

Acute myeloid leukemia (AML) is a complex hematological disease characterized by an uncontrolled proliferation of immature myeloid cells (blasts) which, due to their accumulation in the bone marrow, impair normal hematopoiesis. The incidence of AML increases with age and, despite being the most common type of leukemia in adults, it continues to be associated with the lowest survival rate among all the leukemias [1]. Recent advances have shed light on the pathogenesis of AML, revealing its considerable genetic and clinical heterogeneity, which accounts for the constant need to find new treatment strategies for obtaining satisfying therapeutic outcomes and enhanced quality of life of AML patients [2]. Currently, histone deacetylase inhibitors (HDACi) appear to represent a promising therapy for cancer treatment, and an emerging scenario for AML patients unresponsive to conventional chemotherapy [3,4].

Sirtuins, known as silent information regulator proteins (SIRTs), belong to the group of the NAD^+^-dependent class III histone deacetylases, coming into play in numerous physio–pathological processes, such as the extension of lifespan, age-related disorders, obesity, cardiovascular diseases, neurodegenerative events, and cancer, including leukemias [5]. In this regard, SIRT2, a member of the sirtuin family, has been found to be involved in proliferation and survival of AML cells. This is because mRNA levels of SIRT2 are up-regulated in cells from patients with high-risk AML, where this sirtuin promotes NADPH production by deacetylating the enzyme glucose-6-phosphate dehydrogenase (G6PD) or the phosphorylation of AKT kinase [6,7]. In addition, SIRT2 was also suggested to mediate multidrug resistance of AML cells via ERK1/2 signaling pathways [8]. All this evidence has led SIRT2 to be considered as an unfavorable prognostic biomarker of AML [9] and a new possible target for therapy.

In parallel, an increasing interest in the plant kingdom allowed the identification of new chemical entities for the development of anticancer agents, also in the field of leukemia therapy [10]. Among these, flavonoids, plant secondary metabolites found in vegetables and in a variety of *Citrus* fruits, have gained widespread approval, thanks to their pharmacological properties [11], such as neuroprotective [12,13], antioxidant and anti-inflammatory [14,15], as well as anti-cancer properties [16]. The anti-proliferative, pro-apoptotic and cell cycle-arresting effects of flavonoids contribute to define their anti-leukemic potentiality [17].

In the last decades, *Citrus* × *bergamia* Risso et Poiteau (bergamot), a small evergreen tree belonging to the Rutaceae family, was widely appreciated for its fruits, from which bergamot essential oil (BEO) and bergamot juice (BJ) are obtained. BEO is mainly exploited by the perfume industry and was recently studied for its beneficial effects [18,19]. Contrarily, BJ has long been considered a waste product of the essential oil industry, until it was revaluated by the scientific community, thanks to its notable biological properties, which include being anti-infective [20,21], anti-inflammatory [22] and neuroprotective [23]. The flavonoid fraction of BJ (BJe) appears to play a key role in the anticancer effects of bergamot, as documented in both in vitro and in vivo models [24,25]. Nonetheless, no evidence is currently available on the potential role of BJe in AML management.

Considering this background, our study was designed to investigate the anti-proliferative effect of BJe in human leukemia monocytic THP-1 cells, focusing on the potential involvement of SIRT2 underlying the mechanism of action.

## 2. Materials and Methods

### 2.1. Preparation of a Flavonoid-Rich Extract of Bergamot Juice (BJe)

Bergamot fruits were purchased from the local markets of Reggio Calabria (Italy). After peel removal, fruits were hand-squeezed to remove the primary juice. The residual pulp was processed by a pressing machine to obtain the secondary juice, which was extracted and concentrated, following an SPE extraction by a SupelcleanTM LC-18 SPE cartridge (Supelco Ltd., Bellefonte, PA, USA) according to the procedures described by the seller. The final elution to obtain the flavonoid-rich bergamot fraction was performed with ethanol (utilized as a green solvent) and the eluate was brought to dryness by lyophilization. The obtained powder was stored in the dark at 4 °C prior to use.

### 2.2. Chemical Characterization of BJe

#### 2.2.1. Reagents and Standard Solutions

Neohesperidin, neoeriocitrin, neodiosmin, vicenin-2, rhoifolin and naringin were supplied by Extrasynthèse (Genay, France) and used as standards. The Iso-Disc P-34, 3 mm diameter PTFE membrane (0.45 μm pore size) was from Supelco (Bellefonte, PA, USA). All the other reagents and chemicals employed in this study were of analytical grade and were purchased from Sigma-Aldrich (Milan, Italy).

#### 2.2.2. Sample Preparation

A solution of phosphate saline buffer (PBS)/dimethylformamide (0.9:0.1 *v*/*v*) was used to solubilize the lyophilized powder, reaching the final concentration of 1.25 mg/mL. The obtained solution was centrifuged for 5 min at 3200 rpm. The supernatant was filtered utilizing an Iso-Disc P-34, 3 mm diameter PTFE membrane and separated by reverse phase high performance liquid chromatography (RP-HPLC).

#### 2.2.3. RP-DAD-HPLC Separation and Identification

The separation and identification of compound present in the lyophilized powder were performed using a RP-HPLC with diode array detector (RP-HPLC-DAD) according to Cirmi et al. [13].

#### 2.2.4. Acid Hydrolysis

The acid hydrolysis has been carried out on BJe based on a previously published work [26].

### 2.3. Cell Culture

The human leukemia monocytic THP-1 cell line and human peripheral blood mononuclear cells (PBMCs) were originally obtained from ATCC (Rockville, MD, USA). THP-1 cells were cultured in RPMI 1640 medium with the addition of 10% (*v*/*v*) heat-inactivated fetal bovine serum (FBS), penicillin (100 IU/mL) and streptomycin (100 µg/mL), L-glutamine (2 mM), HEPES (10 mM), sodium pyruvate (1 mM), glucose (2.5 g/L), 2-mercaptoethanol (0.05 mM), at 37 °C in a 5% CO_2_ air humified atmosphere. Each reagent for cell growth was from Gibco (Life Technologies, Monza, Italy). Similarly, PBMCs were cultured in RPMI 1640 supplemented with 10% FBS.

### 2.4. Cell Proliferation Assay

Cell proliferation was determined by the 3-(4,5-dimethylthiazole-2-yl)-2,5-diphenyltetrazolium bromide (MTT) test, as described [27]. Briefly, THP-1 monocytes/PBMCs were seeded in 96-well plates at a density of 5 × 10^4^ cells/well. After 24 h, cells were incubated with fresh medium (for untreated cells) or with medium supplemented with increasing concentrations of BJe (1, 2.5 and 5 mg/mL). After 24, 48 and 72 h, plates were centrifuged to remove supernatants and incubated with phenol red-free fresh medium containing 0.5 mg/mL of MTT (Sigma-Aldrich) at 37 °C for 4 h. Then, the formazan crystals, formed in the wells, were dissolved in 100 µL of a 0.1 N HCl/isopropanol lysis solution. The absorbance of each well was spectrophotometrically measured at a wavelength of 570 nm (reference at 690 nm), using a microplate reader (Bio-Rad Laboratories, Milan, Italy). Results were expressed as percentage of cell viability, compared to untreated cells which were arbitrarily set as 100%. All experiments were performed in eight replicates and repeated three times.

### 2.5. Cell Cytotoxicity Assay

Propidium iodide (PI) exclusion assay was performed to test the cytotoxicity and the membrane integrity after exposure to BJe, as described [28]. Then, THP-1 cells and PBMCs were plated into 24 well-plate (5 × 10^5^ cells/well) and treated with BJe (1, 2.5 and 5 mg/mL) for 24, 48 and 72 h. Afterwards, cells were collected by centrifugation, washed and resuspended in 100 µL of PBS, and then incubated with 10 µL of PI labeling solution (10 µg/mL; Sigma-Aldrich) in darkness at room temperature for 30 min. Dead cells, stained with this DNA intercalating probe, were analyzed by a Novocyte 2000 cytofluorimeter (ACEA Biosciences Inc., San Diego, CA, USA) with FL-2 channel. A minimum of 10,000 events were counted per sample. Percentage of dead cells was calculated versus non-treated cells.

### 2.6. Cell Cycle Analysis

The progression of cells through cell cycle phases was evaluated by PI staining, as described [19]. Briefly, THP-1 cells were seeded in 6-well plates (2 × 10^5^ cells/mL) and, the day after, treated with the BJe (1, 2.5 and 5 mg/mL) for 24, 48 and 72 h. Then, cells were harvested, washed with PBS and fixed with 70% ice-cold ethanol for at least 2 h at 4 °C. Thereafter, cells were washed twice with cold PBS and resuspended in 250 μL of PBS together with 5 μL of RNase A (10 mg/mL; Sigma-Aldrich). After 1 h of incubation at 37 °C, 10 µL of PI (1 mg/mL) were added to samples, which were immediately acquired by Novocyte 2000 cytofluorimeter. Three independent sets of at least 10,000 events were analyzed for each condition.

### 2.7. Assessment of Apoptosis

The involvement of apoptosis was assessed by performing the Annexin V-fluorescein isothiocyanate (FITC)/PI staining, which discriminates from early apoptosis, late apoptosis and necrosis [13]. Briefly, THP-1 cells were seeded in 6-well plates (2 × 10^5^ cells/mL) and, the day after, treated with the BJe (1, 2.5 and 5 mg/mL) for 24, 48 and 72 h. At the end of the treatments, cells were harvested, washed with cold PBS and resuspended in 200 µL of binding buffer 1×, following kit guidelines (BD Biosciences, Milan, Italy). Thereafter, 5 μL of Annexin V-FITC were added to each sample, gently vortexed and incubated in darkness at room temperature for 15 min. After incubation, cells were washed and resuspended in 190 µL of binding buffer plus 10 µL of PI (20 μg/mL). Samples from three independent experiments were analyzed by a Novocyte 2000 flow cytometry, by setting a minimum of 10,000 events for each condition.

### 2.8. Determination of Acetylated P53 Levels in THP-1-Treated Cells

An enzyme-linked immunosorbent assay (ELISA) was performed in THP-1 cells treated for 24 h with BJe (1, 2.5 and 5 mg/mL) or with SIRT2 inhibitors, SirReal2 (10 µM; Selleckchem, Houston, TX, USA) and nicotinamide (NAM, 1 mM; Cayman, Ann Arbor, MI, USA), in order to detect the levels of acetylated p53, employing a commercial kit (Biovision, Milpitas, CA, USA). Briefly, protein concentration from cell lysates was determined using Bio-Rad DC Protein Assay (Bio-Rad Laboratory, Hercules, CA, USA) with bovine serum albumin as standard. Equal amounts of proteins for sample were incubated with 100 µL of biotin-conjugated primary antibody for 1 h at 37 °C in provided strips. After washing, 100 µL of streptavidin HRP-conjugated were added to each well and incubated for an additional 30 min at 37 °C. Then, the plate was incubated with 90 μL of TMB substrate at 37 °C in darkness for further 30 min. Finally, 50 µL of stop solution were added to each well to arrest the color formation and absorbance was read with a microplate spectrophotometer (iMark™ microplate reader, Bio-Rad Laboratories) at 450 nm wavelength. Results were calculated as ratio between values detected in untreated and treated cells.

### 2.9. Cell-Free SIRT2 Histone Deacetylase Activity Assay

SIRT2 activity assay was performed using a SIRT2 direct fluorescent screening assay kit (Cayman Chemical, Ann Arbor, MI, USA), according to the manufacturer’s protocol. Increasing concentrations of BJe (0.01, 0.1 and 1 mg/mL) were tested, employing the recombinant enzyme provided by the kit. The SIRT2 inhibitor, SirReal2 (140 nM), was used as positive control. Briefly, a substrate solution containing 2 mM nicotinamide adenine dinucleotide (NAD^+^) and 125 μM peptide was added to the reaction mixture and incubated for 45 min at 37 °C. Following, a stop/developing solution consisting of developer plus nicotinamide was added to each sample to stop the reaction for 30 min. Thereafter, the emitted fluorescence, index of SIRT2 deacetylase activity, was read using a FLUOstar Omega Plate Reader (BMG LABtech, Ortenberg, Germany) at 350–360 nm excitation wavelength and 450–465 nm emission wavelength.

### 2.10. Real-Time PCR

To quantify the gene expression of SIRT2, p53 (TP53), and caspases-8, -9 and -3 (CASP8, CASP9 and CASP3, respectively), THP-1 cells were seeded in 100 mm Petri dishes (1 × 10^6^ cells/dish) and incubated with fresh medium (untreated cells) or BJe (1, 2.5 and 5 mg/mL), for 6, 12 and 24 h (SIRT2) or for 12 h (TP53, CASP8, CASP9 and CASP3) at 37 °C. Afterwards, RNA extraction from untreated and treated cells was performed employing TRIzol reagent (Invitrogen, Carlsbad, CA, USA), according to the manufacturer’s protocol. An equal amount of total RNA (2 µg) for each sample was reverse transcribed into cDNA employing the High-Capacity cDNA Archive Kit (Applied Biosystems, Life Technologies, Foster City, CA, USA), as previously described [29]. Then, quantitative PCR reaction (qPCR) was carried out in a 96 well-plate, using a 7500 qPCR System (Applied Biosystems), in a total volume of 20 µL, including 1x SYBR^®^ Select Master Mix (Applied Biosystems), 0.2 µM of specific primers and 25 ng of RNA converted into cDNA. Primer sequences used for qPCR are listed in Table 1. Data collected were analyzed using the 2^−∆∆CT^ relative quantification method versus β-actin (ACTB), used as endogenous control. The values are presented as *n*-fold change with respect to untreated cells.

### 2.11. Western Blotting Analysis

For the evaluation of protein expression, THP-1 cells were grown in 100 mm Petri dishes (1 × 10^6^ cells/dish) and exposed to increasing concentrations of BJe (1, 2.5 and 5 mg/mL) for 24 h. At the end of treatments, cells were harvested, washed with PBS and lysed using RIPA buffer (Sigma-Aldrich), supplemented with 1% cocktail of protease and phosphatase inhibitors (Sigma-Aldrich). The lysed cells were centrifuged at 12,000× *g* for 15 min at 4 °C and supernatant was collected. For each sample, the protein concentration of supernatant was determined using Bio-Rad DC Protein Assay (Bio-Rad Laboratory) and bovine serum albumin as standard. Equal amounts of proteins (30 μg/lane) were separated by 10% sodium dodecyl sulphate–polyacrylamide gel electrophoresis (SDS-PAGE) and electro-transferred on a polyvinylidene fluoride (PVDF; GVS Life Sciences, ME, USA) or nitrocellulose membranes (Merck Millipore, Darmstadt, Germany), where non-specific binding sites were blocked with 5% (*w*/*v*) non-fat dry milk for 1 h at room temperature. Thereafter, membranes were incubated overnight at 4 °C with the following primary antibodies: rabbit monoclonal anti-cleaved caspase-8, anti-caspase-9, anti-caspase-3, anti-poly ADP ribose polymerase (PARP), anti-phospho-AKT and anti-AKT, all diluted 1:1000 in milk or BSA and purchased from Cell Signaling Technology (Danvers, MA, USA). Mouse monoclonal anti-p53 (1:200) was purchased by Thermo-Fisher Scientific (Rockford, IL, USA). Similarly, mouse monoclonal anti-β-actin-peroxidase (1:50,000) was from Sigma-Aldrich. Then, membranes were washed thrice in Tris-buffered saline containing 0.15% of Tween 20 (TBST) and incubated with horseradish peroxidase-conjugated goat anti-mouse or anti-rabbit IgG secondary antibodies (1:5000, Sigma-Aldrich) for 2 h at room temperature. Chemiluminescence of protein bands was obtained using Luminata Forte Western HRP Substrate (Merck Millipore) and visualized by a chemiluminescent detection system C-Digit Blot Scanner (Li-COR Bioscience, Lincoln, NE, USA). Protein bands were quantified using Image Studio software (Li-COR Bioscience). The β-actin was used as housekeeping protein.

### 2.12. Statistical Analysis

Data from three sets of experiments performed in triplicate were expressed as mean ± standard error of the means (SEM). They were statistically evaluated for differences using one-way analysis of variance (ANOVA), followed by the Dunnett’s multiple comparison test (GraphPad Prism Software for Science, San Diego, CA, USA). *p*-values less than or equal to 0.05 were considered significant.

## 3. Results

### 3.1. Chromatographic Analysis of BJe

**Figure 1:**
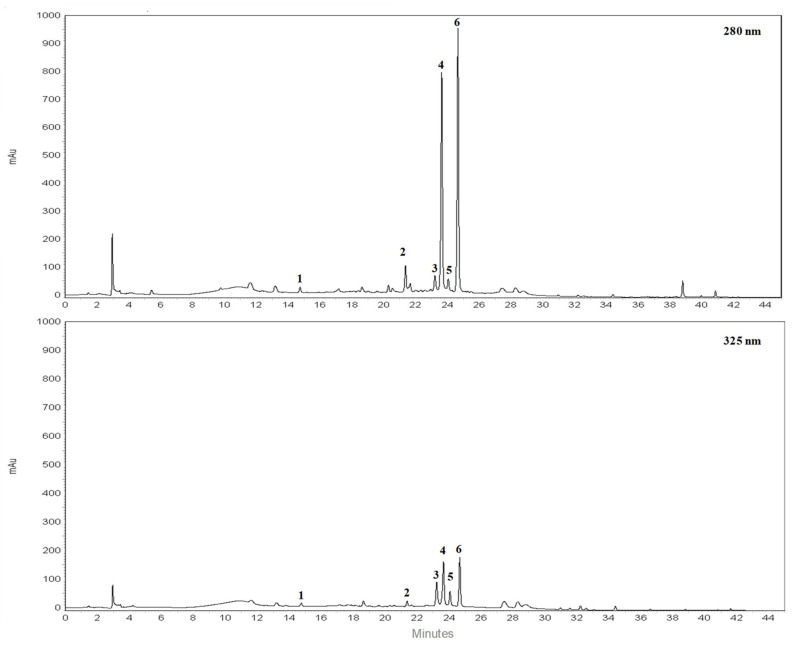
Representative chromatograms of flavonoid composition of BJe. Reverse phase diode array detector high performance liquid chromatography (RP-HPLC-DAD) separation of compounds present in the lyophilized powder recorded at 280 nm and 325 nm. Flavonoids corresponding to peaks 1–6, expressed in milligrams (mg) per milliliter (mL) of liquid extract, are the following: vicenin-2 (**1**), neoeriocitrin (**2**), rhoifolin (**3**), naringin (**4**), neodiosmin (**5**) and neohesperidin (**6**). Peak identification was performed by comparing retention time, UV spectra and spiking the samples with pure reference compounds.

RP-HPLC-DAD separation was carried out to identify compounds present in the lyophilized powder. The preliminary analysis of chromatographic separation performed at 280 and 325 nm allowed us to discriminate between flavanone and flavone basic skeletons present in the extract. The two chromatograms reported in the Figure 1 showed a prevalence of compounds 2, 4, 6 possessing a flavanone skeleton than compounds 1, 3, 5 belonging to the flavone class. The treatment with aqueous HCl revealed that compounds 2, 4, 6 are not resistant to acid hydrolysis, suggesting the presence of O-linked saccharide moieties in their aglycone structure (data not shown). By comparing the retention time, UV spectra and spiking of these compounds with pure reference compounds, three main peaks of chromatogram were identified as neoeriocitrin (2), naringin (4) and neohesperidin (6). In particular, neohesperidin and naringin represent, by far, the most abundant flavonoids of BJe (0.59 ± 0.037 and 0.44 ± 0.017 mg/mL respectively), since they account for about 83% than whole amount of extract employed to perform the analysis, followed by neoeriocitrin (0.11 ± 0.011 mg/mL). The other three compounds (1, 3, 5) showed UV spectra with maxima of absorption in the 240–280 nm range (called band II) and additionally in the 300–380 nm range (called band I). Compound 1 demonstrated resistance to acid hydrolysis, suggesting the presence of C-linked saccharide moieties in its aglycon structure, while compounds 3 and 5 were not resistant to acid hydrolysis. Therefore, based on the retention time, UV spectra and spiking of the flavones 1, 3, 5 with commercial standards, the remaining three peaks of the chromatogram were identified as vicenin-2 (1), rhoifolin (3) and neodiosmin (5). They were present in an amount well below 0.1 mg/mL. The quantitative determination of flavonoids identified in BJe was depicted in Table 2.

### 3.2. BJe Inhibited the Growth of Human Leukemia Monocytic THP-1 Cells

**Figure 2:**
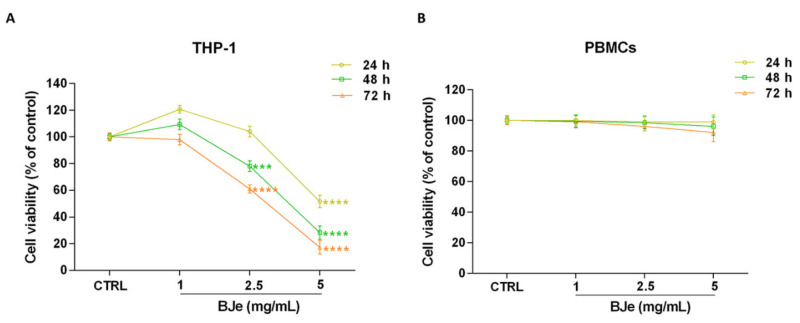
Effect on cell proliferation of BJe in THP-1 cells and PBMCs. Both cell lines were exposed to different concentrations of BJe (1–5 mg/mL) for 24, 48 and 72 h. Cell viability rate was assessed by MTT test (**A**,**B**). Results are expressed as percentages ± SEM of absorbance compared to values detected in the untreated cells (control, CTRL). Each concentration was tested eight-fold, and three independent experiments were carried out (*n* = 24). *** *p* < 0.001 and **** *p* < 0.0001 vs. CTRL.

To investigate the effects of BJe on cell proliferation, the MTT test was performed. Figure 2A shows that treatment with BJe was able to hamper THP-1 cell proliferation at the reported times of incubation. After 24 h, only 5 mg/mL of BJe induced a significant decrease of cell proliferation (−48.4 ± 4.6%; *p* < 0.0001 vs. CTRL). Instead, despite to different extent, both 2.5 and 5 mg/mL of BJe significantly decreased THP-1 cell growth, after 48 h (−22 ± 4%, *p* < 0.001 and −71.7 ± 5%, *p* < 0.0001, vs. CTRL, respectively) and 72 h (−39 ± 3%, *p* < 0.0001 and −82.8 ± 5%, *p* < 0.0001, vs. CTRL, respectively) of treatment, reaching an IC_50_ of 2.92 ± 0.32 mg/mL at the latter time point. The concentration of 1 mg/mL did not impair cell growth at any time tested (Figure 2A). Noteworthy, BJe did not significantly alter the proliferation of normal human PBMCs at the same times and concentrations tested in cancer cells (Figure 2B).

### 3.3. BJe Induced Cell Death in THP-1 Cells

**Figure 3:**
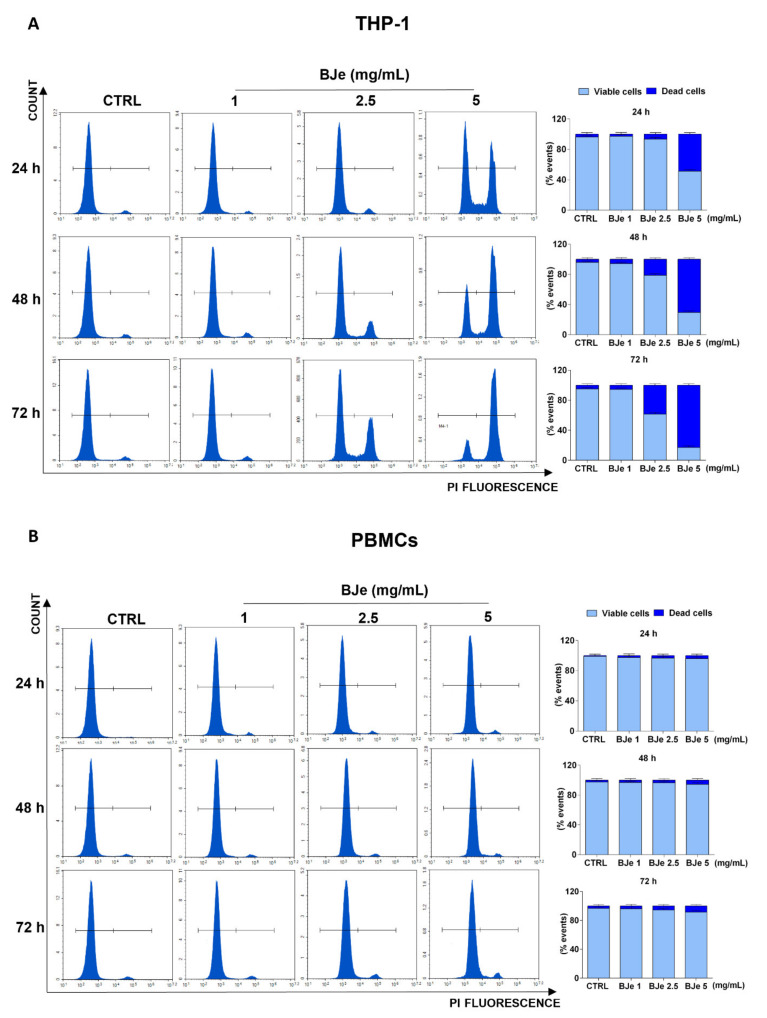
Cytotoxicity evaluation of BJe in THP-1 cells and PBMCs. Both cell lines were exposed to different concentrations of BJe (1–5 mg/mL) for 24, 48 and 72 h. Cytotoxic activity of BJe was evaluated by PI staining through flow cytometry (**A**,**B**). On the left, representative plots of three different experimental sessions of PI staining performed in triplicate (*n* = 9) are displayed, where the first peak represents viable cells, while the second one dead cells. On the right, histograms report the percentage of viable (PI^−^) and dead (PI^+^) cells ± SEM of three independent experiments performed in triplicate (*n* = 9).

At this point, we assessed the potential cytotoxic effect underlying the above documented anti-proliferative activity of BJe, by PI staining. As shown in Figure 3A, the 5 mg/mL concentration induced a cytotoxic effect in THP-1 cells (48.6 ± 2%, *p* < 0.0001 vs. CTRL) already after 24 h of exposure, whereas the BJe 2.5 mg/mL induced cell death (21.2 ± 3.1%, *p* < 0.001 vs. CTRL) only after 48 h. Conversely, BJe 1 mg/mL did not exert any cytotoxicity in THP-1 cells (Figure 3A), corroborating the outcome obtained by MTT assay. Again, BJe did not cause any significant increase in PBMCs cell death at any of the timings or concentrations evaluated (Figure 3B).

### 3.4. BJe Induced a Blockage of Cell Cycle in THP-1 Cells

**Figure 4:**
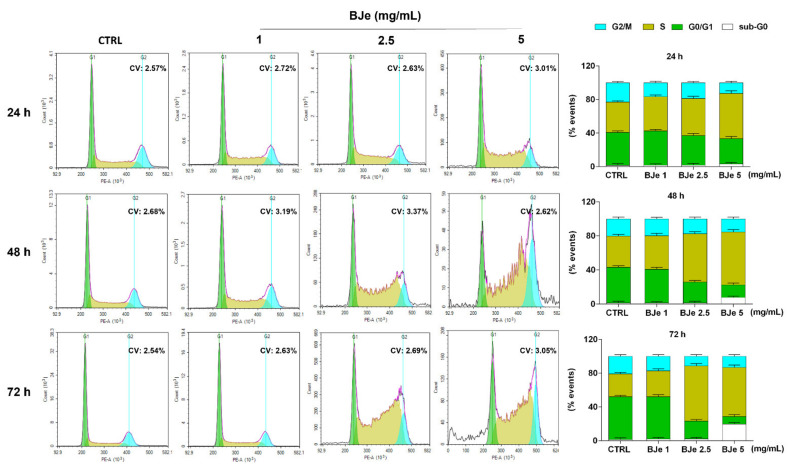
Influence of BJe on cell cycle distribution of THP-1 cells. Progression of THP-1 cells through the cell cycle phases was assessed by cytofluorimetric analysis after exposure to BJe (1–5 mg/mL) for 24, 48 and 72 h. Representative plots of three different experimental sessions of PI staining performed in triplicate (*n* = 9) are shown (on the **left**), along with the coefficient of variation (CV) value. Histograms report the percentage of cells present in sub G0 (white bar), G0/G1 (green bar), S (dark yellow bar), and G2/M (cyan bar) phases, expressed as the mean ± SEM of three independent experiments performed in triplicate (*n* = 9) (on the **right**).

Following, we investigated whether the anti-proliferative activity of BJe may be linked to its potential capacity to influence the THP-1 cell cycle distribution. Figure 4 shows that the treatment with 2.5 and 5 mg/mL of BJe increased the cell population in S phase (up of 38.3 ± 3% and 31.2 ± 2.8% vs. CTRL after 72 h, respectively), thus decreasing the number of cells in G0/G1 one (down of 29.7± 1.9% and 41.7± 2% vs. CTRL after 72 h, respectively), as well as those in G2/M one (down of 9.4 ± 2% and 7.7 ± 2.2% vs. CTRL after 72 h, respectively). Notably, a modulation of the cell cycle was appreciated already after 24 h of exposure to BJe 5 mg/mL and after 48 h to BJe 2.5 mg/mL, reaching a more evident S phase arrest at longer times. The 1 mg/mL concentration demonstrated not to be able to alter the cell cycle of THP-1 cells at any of the tested timings (Figure 4).

### 3.5. BJe Induced Apoptosis in THP-1 Cells

**Figure 5:**
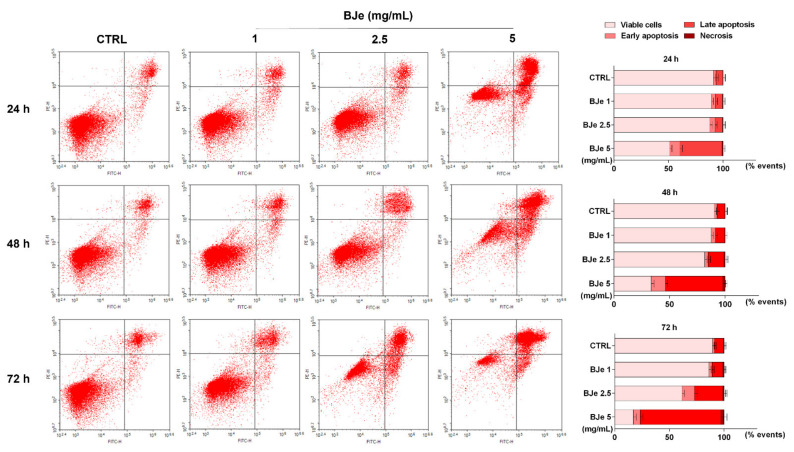
Cytofluorimetric evaluation of apoptosis in THP-1 cells exposed to BJe. The evaluation of apoptosis was performed by the Annexin V-FITC/PI assay. On the left, representative Annexin V vs. PI dot plots of the THP-1 cells treated with different concentrations (1–5 mg/mL) of BJe for the indicated times are displayed. Lower left quadrant contains the viable cells (Annexin V^−^/PI^−^), lower right quadrant contains the cells in early apoptosis (Annexin V^+^/PI^−^), upper right quadrant contains the cells in late apoptosis (Annexin V^+^/PI^+^), while upper left quadrant contains the necrotic cells (Annexin V^−^/PI^+^). On the right, histograms report the percentages of cells for each quadrant ± SEM of three independent experiments performed in triplicate (*n* = 9).

In order to evaluate if apoptosis was implied in the cell death induced by BJe in THP-1 cells, the Annexin V-FITC/PI cytofluorimetric assay was performed. As shown in Figure 5, treatment with 2.5 and 5 mg/mL of BJe for 24 h increased the percentage of cells undergoing apoptosis (both in early and late) up to 11.6 ± 2.1% and 47.8 ± 2.3%, respectively. After 48 h, both 2.5 and 5 mg/mL of BJe induced apoptosis up to 17.5 ± 2.1% and 64.4 ± 2.2%, respectively, and up to 37.3 ± 2.5% and 80 ± 2.5% after 72 h, respectively. Finally, according to cell viability assays, the 1 mg/mL concentration of BJe did not trigger apoptosis at any of the tested timings (Figure 5).

### 3.6. BJe Activated Both Extrinsic and Intrinsinc Apoptotic Cascade

**Figure 6:**
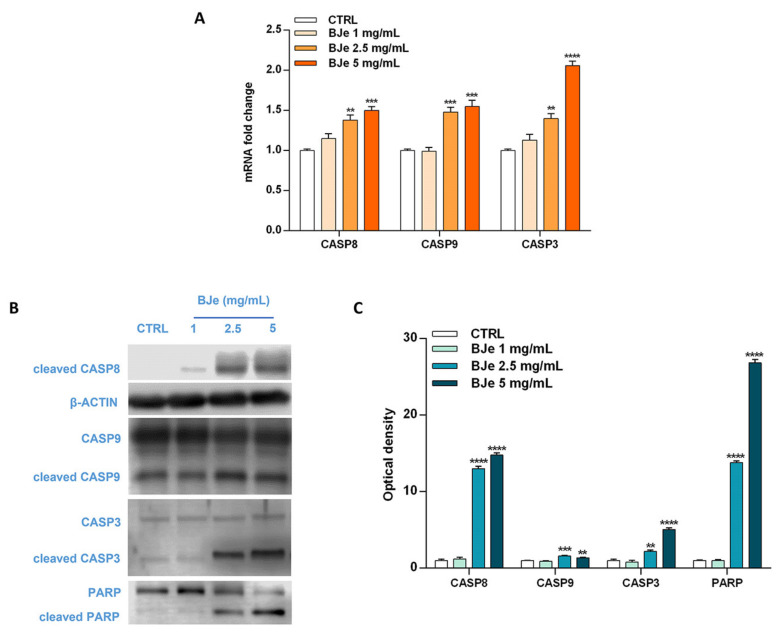
Gene and protein expression of apoptosis-related factors modulated by BJe in THP-1 treated cells. Cells were exposed to different concentrations (1–5 mg/mL) of BJe for 12 h and 24 h and were processed for gene and protein expression studies, respectively. The gene expression was calculated by the 2^–ΔΔCT^ method, with β-actin as housekeeping gene. Results are expressed as fold change compared to the untreated cells (**A**). A representative immunoblot of three independent experiments is shown (**B**). Densitometric analysis of bands from three independent blots is presented (**C**). Results are expressed as ratio of the cleaved form with respect to the relative zymogen (cleaved CASP9/CASP9, cleaved CASP3/CASP3 and cleaved PARP/PARP), while the expression of cleaved CASP8 was normalized against β-actin. Protein levels are extrapolated as values detected in the untreated cells (CTRL), which are arbitrarily assigned as 1. Data, represented as mean ± SEM, report the values obtained in three different sets of experiments for Western blotting (*n* = 3), while made in triplicate for RT-PCR (*n* = 9). ** *p* < 0.01, *** *p* < 0.001 and **** *p* < 0.0001 vs. CTRL.

Based on the Annexin V-FITC/PI staining assay results, we focused on the molecular mechanisms underlying the programmed cell death to ascertain whether BJe triggered an extrinsic or intrinsic apoptotic pathway. For this reason, we evaluated the cleavage of caspases, known for their pivotal role in this process, by Western blotting, as well as their gene expression by RT-PCR. Interestingly, as shown in Figure 6A, both 2.5 and 5 mg/mL BJe significantly increased gene expression of CASP8 by 1.38 ± 0.06-fold and 1.5 ± 0.05-fold (*p* < 0.01 and *p* < 0.001 vs. CTRL), respectively. BJe also augmented CASP9 mRNA levels by 1.48 ± 0.06-fold and 1.55 ± 0.08 (for both *p* < 0.001 vs. CTRL), respectively, while CASP3 by 1.4 ± 0.06-fold and 2.06 ± 0.05-fold (*p* < 0.01 and *p* < 0.0001 vs. CTRL), respectively (Figure 6A). BJe significantly promoted the cleavage of both caspase-8 and -9 proteins, initiators of extrinsic and intrinsic apoptotic pathway, respectively. In detail, the exposure of THP-1 cells to 2.5 and 5 mg/mL enhanced the levels of cleaved caspase-8 of 13 ± 0.29-fold and 14.8 ± 0.29-fold (for both *p* < 0.0001 vs. CTRL), as well as cleaved caspase-9 of 1.58 ± 0.08-fold and 1.37 ± 0.07-fold (*p* < 0.001 and *p* < 0.01 vs. CTRL). Consequently, caspase-3 cleavage was observed at BJe 2.5 mg/mL and 5 mg/mL concentrations (2.2 ± 0.19-fold, *p* < 0.01, and 5.05 ± 0.25-fold, *p* < 0.0001 vs. CTRL, respectively), which in turn activated PARP, downstream effector of caspase-3, with a significant increase of its cleaved form (13.8 ± 0.22-fold for BJe 2.5 mg/mL, *p* < 0.0001 and 26.8 ± 0.45-fold for BJe 5 mg/mL, *p* < 0.0001 vs. CTRL; Figure 6B,C). Therefore, the activation of the two caspases proved that BJe induced THP-1 cell death by initiating both extrinsic (caspase-8) and intrinsic (caspase-9) apoptotic pathways.

### 3.7. BJe Increased Levels of Acetylated p53 in THP-1 Cells

**Figure 7:**
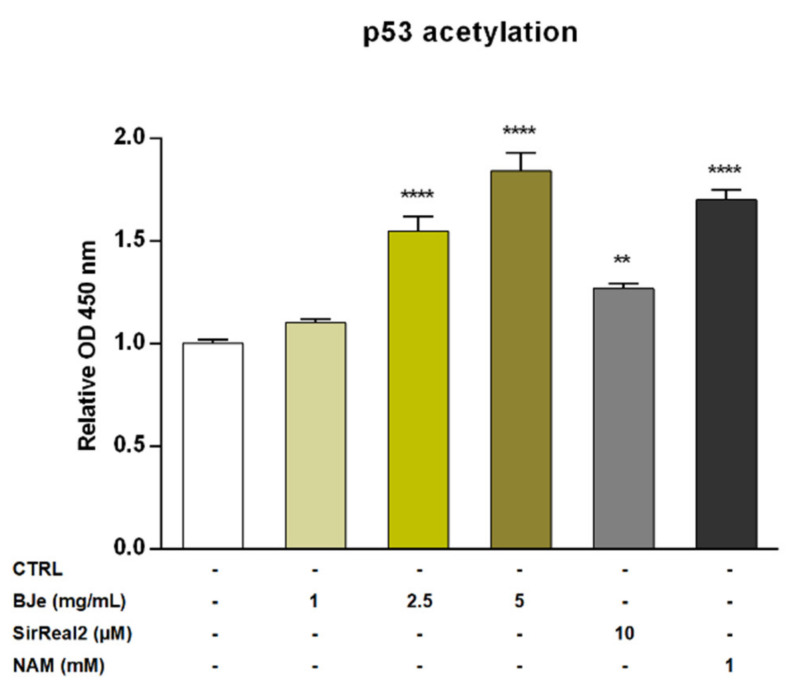
Acetylation of p53 in THP-1 cells after treatment with BJe for 24 h. Control (CTRL, white bar) consists in untreated cells, while BJe (yellow bars) was assessed at the concentrations shown in the figure. Specific and non-specific SIRT2 inhibitors, SirReal2 (grey bar) and NAM (black bar), were used as positive controls. Relative protein levels of acetylated p53 were quantified by ELISA. Data are expressed as fold change relative to controls (untreated cells) and represent the mean ± SEM of three independent experiments performed in triplicate (*n* = 9). ** *p* < 0.01 and **** *p* < 0.0001 vs. CTRL.

Recently, SIRT2 was depicted as an unfavorable prognostic marker of AML [9]. For this reason, we investigated the potential modulatory effect of BJe on the SIRT2 enzyme by quantifying the levels of acetylated p53 protein, a well-known SIRT2 substrate. The exposure of THP-1 cells to 1 mg/mL concentration of BJe for 24 h did not alter levels of acetylated p53. Conversely, both 2.5 and 5 mg/mL of BJe significantly increased p53 acetylation (1.55 ± 0.07-fold, *p* < 0.0001, and 1.84 ± 0.09-fold, *p* < 0.0001, vs. CTRL, respectively; Figure 7). In addition, to ensure a reliable comparison of these results, we used a specific (SirReal2, 10 µM) and a non-specific (nicotinamide, NAM, 1 mM) SIRT2 inhibitors, as positive controls. Similar to BJe, both inhibitors significantly induced an increase of levels of acetylated p53 with SirReal2 up to 1.27 ± 0.03-fold and NAM up to 1.70 ± 0.05-fold (*p* < 0.01 and *p* < 0.0001 vs. CTRL, respectively), suggesting that BJe acted as a SIRT2 inhibitor.

### 3.8. BJe Reduced SIRT2 Activity in the Isolated Recombinant Enzyme

**Figure 8:**
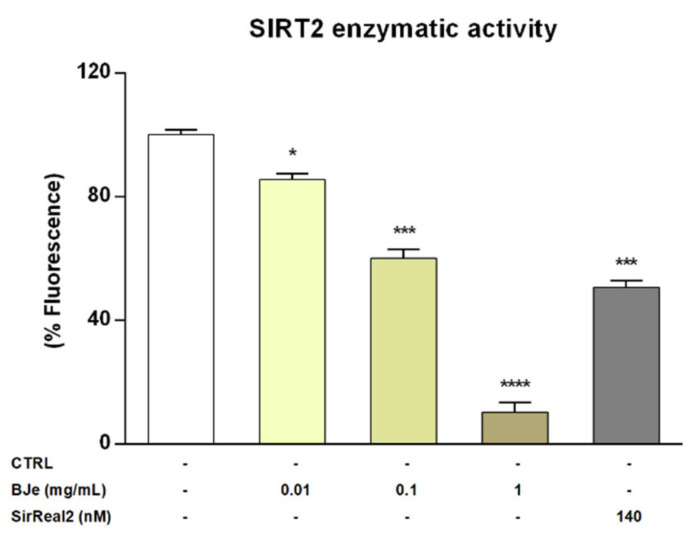
SIRT2 activity in a cell-free model. Control (CTRL, white bar) consists in the isolated recombinant enzyme, while BJe (yellow bars) was tested at the concentrations shown in the figure. The SIRT2 inhibitor, SirReal2, was employed as positive control (grey bar). Data are expressed as percentage of fluorescence corresponding to SIRT2 enzymatic activity ± SEM of three independent experiments performed in triplicate (*n* = 9). * *p* < 0.05, *** *p* < 0.001 and **** *p* < 0.0001 vs. CTRL.

Given the encouraging results obtained in vitro, we wanted to verify whether BJe could directly inhibit the enzymatic activity of SIRT2. To this purpose we assayed its activity employing a cell-free model, consisting of the isolated recombinant enzyme. Interestingly, results of these experiments showed that BJe, at each concentration tested, was able to significantly inhibit the deacetylase activity of SIRT2, confirming the outcomes obtained in vitro (Figure 8). In particular, BJe displayed the strongest inhibitory effect at a concentration of 1 mg/mL, reaching a decrease of 89.9 ± 3% (*p* < 0.0001, vs. CTRL). Moreover, the 0.1 mg/mL concentration significantly reduced the enzymatic activity of 40 ± 3% (*p* < 0.001 vs. CTRL), whereas BJe 0.01 mg/mL showed the weakest inhibitory activity against SIRT2 (14.6 ± 2%, *p* < 0.05 vs. CTRL). Therefore, the calculated IC_50_ value for BJe was 0.13 ± 0.01 mg/mL. The specific SIRT2 inhibitor SirReal2 was employed at the IC_50_ concentration (140 nM), as a positive control.

### 3.9. BJe Modulated the Expression of SIRT2 Gene in THP-1 Cells

**Figure 9:**
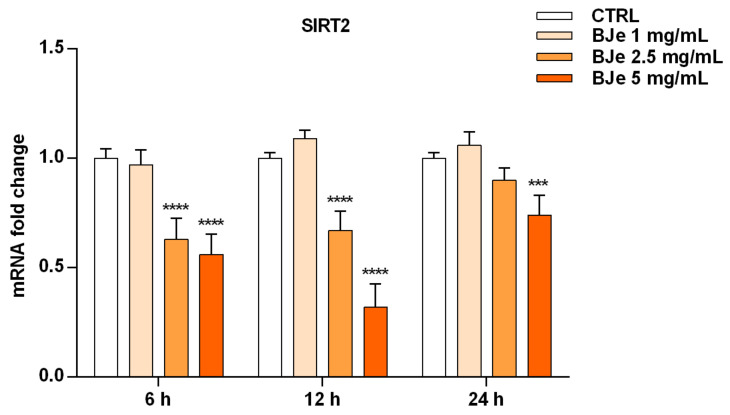
Gene expression of SIRT2 modulated by BJe in treated THP-1 cells. Cells were exposed to different concentrations of BJe (1–5 mg/mL, orange bars) for 6, 12 and 24 h. The mRNA levels were quantified by RT-PCR analysis and calculated by using the 2^–ΔΔCT^ method, with β-actin as housekeeping gene. Results are expressed as fold change compared to the untreated cells. Data, represented as mean ± SEM, report the values obtained in three different sets of experiments made in triplicate (*n* = 9). *** *p* < 0.001 and **** *p* < 0.0001 vs. CTRL.

After ascertaining the capability of BJe to inhibit SIRT2 enzymatic activity in both cell-based and cell-free settings, we wondered whether this could be also due to an alteration of SIRT2 gene expression induced by BJe in THP-1 cells (Figure 9). Worthy of note, the results of RT-PCR analysis showed a significant lowering of SIRT2 gene level compared to untreated cells of 1.6 ± 0.06-fold down with 2.5 mg/mL, and of 1.8 ± 0.05-fold down with 5 mg/mL of BJe (for both *p* < 0.0001 vs. CTRL), already after 6 h of treatment. After 12 h of incubation with BJe, the inhibitory effect on SIRT2 gene expression was almost unchanged with the 2.5 mg/mL (1.49 ± 0.05-fold down, *p* < 0.0001 vs. CTRL) and was more marked with the 5 mg/mL (3.12 ± 0.06-fold down, *p* < 0.0001 vs. CTRL). This early modulation of SIRT2 mRNA levels by BJe was attenuated after 24 h, staying significant only for the 5 mg/mL. Again, no significant modulation of SIRT2 levels was observed with 1 mg/mL concentration, at any of the tested timings (Figure 9).

### 3.10. BJe Modulated the SIRT2/AKT/p53 Pathway

**Figure 10:**
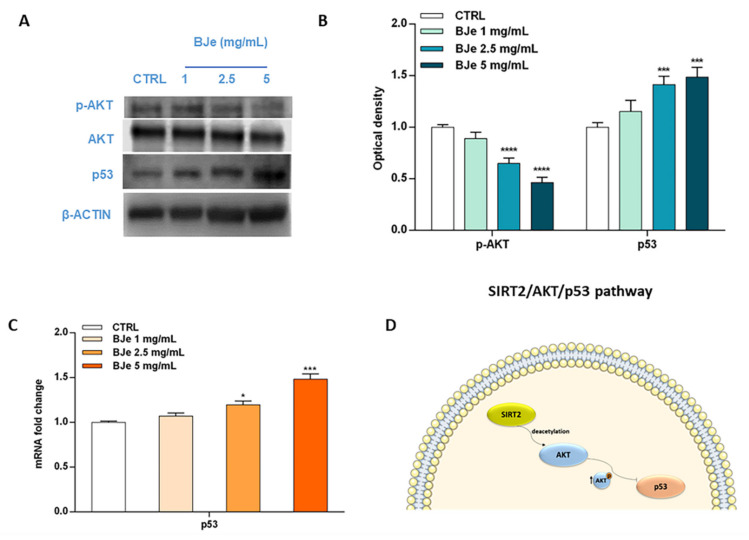
Phosphorylation of AKT along with gene and protein expression of p53 modulated by BJe in THP-1 treated cells. Cells were exposed to different concentrations (1–5 mg/mL) of BJe for 12 h and 24 h, prior being processed for gene analysis by RT-PCR as well as for protein expression studies by Western blotting, respectively. A representative immunoblot of three independent experiments is shown (**A**). Densitometric analysis of bands from three independent blots is presented (**B**). Results are expressed as ratio of phosphorylated form with respect to total protein (p-AKT/AKT), while the expression of p53 was normalized against β-actin. Protein levels are extrapolated as values detected in the untreated cells (CTRL), which are arbitrarily assigned as 1. The gene expression of p53 was quantified by RT-PCR analysis and calculated by the 2^–ΔΔCT^ method, with β-actin as housekeeping gene. Results are expressed as fold change compared to the untreated cells (**C**). Data, represented as mean ± SEM, report the values obtained in three different sets of experiments for Western blotting (*n* = 3), while made in triplicate for RT-PCR (*n* = 9). * *p* < 0.05, *** *p* < 0.001, and **** *p* < 0.0001 vs. CTRL. The putative mechanism of action of BJe involving SIRT2/AKT/p53 pathway is depicted (**D**).

Acknowledging the relevance of the crosstalk between the kinase AKT and p53 in the triggering of apoptosis, as well as the role of SIRT2 in its modulation, we then hypothesized that BJe could alter this pathway to elicit its pro-apoptotic effects (Figure 10). Western blotting data indicated a significant modulation of these factors by BJe (Figure 10A,B). In detail, the phosphorylation levels of AKT significantly decreased in response to BJe after 24 h of incubation, whereas total AKT protein levels kept constant throughout the course of the experiment. This occurred both at 2.5 and 5 mg/mL concentrations, where p-AKT levels lowered of 1.54 ± 0.03-fold and of 2.17 ± 0.03-fold (for both *p* < 0.0001, vs. CTRL), respectively. Results on p53 demonstrated that BJe was able to modulate the expression of p53 both at gene and at protein level. In the latter case, there was a significant growth in the protein amount with 2.5 mg/mL (of 1.41 ± 0.04-fold) and 5 mg/mL (of 1.50 ± 0.05-fold) concentrations of BJe (for both *p* < 0.001 vs. CTRL). The results of p53 protein expression reflected and strengthened data obtained from RT-PCR analysis (Figure 10C). Therefore, these outcomes corroborate our assumption that BJe induced apoptosis via inhibiting SIRT2 activity, which in turn decreased the activation of AKT and consequently augmented p53 activity, thus prompting cells to undergo apoptosis (Figure 10D).

## 4. Discussion

The link between nutrition and cancer has garnered an ever-growing interest for the protective effects of plant-derived natural compounds, commonly found in the diet. Among the sources of these valuable molecules, *Citrus* fruits (CF, i.e., oranges, lemons, limes, bergamots, grapefruits, and tangerines), which are mainly consumed in the Mediterranean diet, stand out among the others [30]. Regarding cancer, the scientific community has long supported their potentiality as anti-tumor agents, hence suggesting *Citrus* extracts and derivatives as co-adjuvants in cancer therapy [31,32]. This is because CF represent the main dietary source of flavonoids which are shown to interfere with the process of carcinogenesis by hampering multiple signal transduction pathways, by counteracting proliferation, angiogenesis, metastasis or by promoting apoptotic mechanisms [33]. All these properties underlie the antileukemic activity of flavonoids observed both in vitro [34] and in vivo [35]. In the field of AML, continuous efforts are focused on finding new therapeutic approaches with high efficacy and few side effects. Thereby, the role of natural products targeting leukemic cells is being considered a possible help for AML management [36]. On this line, the *Citrus* flavonoid luteolin was shown to inhibit the growth of MOLM-13 and MV4–11 AML cells by down-regulating eIF4E phosphorylation and arresting the cell cycle in G0/G1 phase [37]. Again, nobiletin was demonstrated to induce antileukemic effects through the down-regulation of c-KIT gene in THP-1 cells [38], while treatment with diosmetin delayed tumor growth in AML mouse xenografts [39].

In recent years, *Citrus* *× bergamia* proved to possess anti-cancer properties, among others. We demonstrated that BJ was able to reduce the growth rate of human neuroblastoma SH-SY5Y cells by inducing a cell cycle block in G1 phase and a loss of adhesive capacity [40]. This latter appeared responsible for its anti-migratory effect, leading to the reduction of lung metastasis colonization in a model of spontaneous neuroblastoma metastasis formation in SCID mouse [24]. In parallel, BJ was also shown to inhibit the growth of human hepatocellular carcinoma HepG2 cells by acting on p53, p21 and NF-κB pathways [41]. Afterwards, we focused on the flavonoid-rich extract of BJ, namely BJe, to study its antitumor activity. In vitro, it inhibited the growth of human colorectal carcinoma HT-29 cells by multiple mechanisms, including the increase of reactive oxygen species production, the fall of the mitochondrial membrane potential and oxidative damage to DNA at high concentrations, whereas the inhibition of MAPKs pathways and the modulation of apoptosis- and cell cycle-related proteins occurred at low concentrations [42]. In vivo, BJe was able to prevent spontaneous tumorigenesis in Pirc rats (F344/NTac-Apc^am1137^), a genetic model of colorectal cancer, through a mechanism linked to its anti-inflammatory and pro-apoptotic properties [25]. Acknowledging the anti-cancer activity of BJe documented in solid tumors and the current evidence on antileukemic potentiality of flavonoids, we investigated, for the first time, the role of BJe in the field of hematological diseases, such as AML.

The quali-quantitative determination of flavonoids present in BJe identified neohesperidin and naringin among the most representative components. Although both compounds have been claimed not to be effective in inducing anti-proliferative effects in THP-1 cells at micromolar concentrations [43], this did not occur when we employed the extract. Indeed, the high-water solubility of BJe allowed us to use high extract concentrations that corresponded to testing neohesperidin and naringin in the millimolar range. This appears to explain our strong results, bearing in mind that, in our extract, neohesperidin and naringin were together with other flavonoids that participated in the anti-proliferative effects we observed, probably due to synergistic interaction. Interestingly, the anti-proliferative effects of BJe observed in THP-1 cells did not occur in primary PBMCs, suggesting the safety of our extract.

Mechanistically, natural compounds were demonstrated to inhibit proliferation, migration, and tumor progression by inducing cell cycle arrest and apoptosis or autophagy in leukemia cells [36]. On this line, we investigated the mechanism underlying the anti-proliferative effect of BJe by cell cycle and apoptosis studies. In AML, recurrent alterations during S phase cause an accelerated and enhanced replication of genome, that in turn increases cell proliferation and makes cells vulnerable to acquiring mutations, thus limiting the efficacy of chemotherapies [44]. Interestingly, in our work we witnessed an accumulation of cell population in S phase, thus demonstrating the influence of BJe on cell cycle progression of THP-1 cells. At least in part, the block of the cell cycle may be responsible for the pro-apoptotic effect of BJe, that may be also due to the interaction with specific factors linked to apoptosis. This process can be conducted in two distinct yet interconnected signaling pathways, both including the activation of cysteine aspartyl proteases, called caspases. The extrinsic pathway, mediated by caspase-8, describes an apoptotic event triggered by extracellular stimulations, which are recognized and propagated by specific membrane receptors. The intrinsic pathway, originated in mitochondria, can be triggered by multiple intracellular stimulations, through the involvement of caspase-9. Both pathways determine the cleavage of the downstream executioner proteins (i.e., caspase-3), followed by the cleavage of PARP [45]. Interestingly, BJe was able to target both receptor- and mitochondria-mediated apoptosis, as witnessed by the increased cleavage of caspase-8 and -9, respectively. These results reflected both at gene and protein levels. This brought the consequent cleavage of caspase-3 and that of PARP, thus unleashing apoptosis by targeting both extrinsic and intrinsic pathways contemporarily. Notably, this occurs also for the pro-apoptotic effect of BJe in HepG2 cells [41].

In the last decade, SIRTs have been extensively studied as potential molecular targets in several age-related diseases. Of note, small molecules including some flavonoids appeared to be effective SIRT modulators [46]. Previously, we have investigated the role of BJe and of its flavonoids against SIRT1, one of the seven isoforms of the sirtuin family, in an in vitro model of inflammation, observing a modulation of this sirtuin [47]. In tumorigenesis, the SIRT2 enzyme is known to exert a dual role, acting both as a tumor promoter and suppressor [48]. In AML, recent studies have indicated the key role of SIRT2 as a proliferation marker [6,7]. SIRT2 is a histone deacetylase primarily placed in the cytoplasm which transiently migrates in the nucleus during mitosis, by reducing the acetylation level of some substrates (i.e., p53, α-tubulin, FOXOs, NF-κB, PEPCK1 etc.). Consistent with this, very recently, we found that flavanones neohesperidin and naringin, along with their aglycones, inhibited SIRT2 deacetylase activity [49]. Therefore, in our study, we evaluated, for the first time, the possible implication of SIRT2 in the anti-leukemic effect mediated by BJe in THP-1 cells. Since p53 is a target for SIRT2 deacetylation [50], we quantified the level of acetylated p53 in BJe-treated THP-1 cells, experiencing its significant increase. Hence, the high p53 acetylation, meaning its higher activity, suggested an in vitro SIRT2 inhibition mediated by BJe. In addition, the use of SirReal2 and NAM, both SIRT2 inhibitors, further supported the inhibitory effect on SIRT2 by our extract. In particular, BJe showed somewhat higher effect than SirReal2, a SIRT2 specific inhibitor, and comparable to NAM, that is a SIRT2 a-specific inhibitor. This could suggest that these high concentrations of flavonoids present in BJe might simultaneously inhibit more sirtuins causing THP-1 cell death via the increase of p53 acetylation, as suggested by Peck et al. [51]. Our in vitro results were strengthened by the abiotic assay performed with the isolated enzyme, thus suggesting that BJe inhibits SIRT2 activity also by direct targeting this sirtuin.

Since the inhibition of enzymes can be either achieved by hampering the gene expression or by the enzymatic activity, we wondered whether, aside from a modulation of the deacetylase activity, the mRNA levels of SIRT2 could also be influenced by our extract. Therefore, we quantified them in THP-1 treated cells, finding an early reduction induced by BJe, already after 6 h of treatment. Notably, the lowering effect on SIRT2 gene expression attenuated after 24 h of incubation with extract. This could be due to the activation of a response by cancer cells, like THP-1, which counteracted SIRT2 down-regulation mediated by BJe, by restoring its mRNA at the level of untreated cells.

Moreover, it is known that AKT, a serine/threonine kinase, plays an important role in anti-apoptotic signaling, targeting proteins such as BAD, caspase-9 and p53 [52,53], thus being implicated in several aggressive human cancers, including AML [54]. In addition, some previous data documented a specific interplay between SIRT2 and AKT [6,55]. Indeed, SIRT2 deacetylase activity positively regulates the binding of AKT to inositol 1,4,5-triphosphate (PI3K), leading to the activation of the PI3K/AKT pathway, involved in cell proliferation, survival, and apoptosis. In addition, the overexpression of SIRT2 was significantly associated with an increase of AKT phosphorylation in PADI3-expressing HCT116 colon cancer cells [56]. Interestingly, one of the most abundant flavonoids in *Citrus* fruits, naringenin, was shown to induce apoptosis in human leukemia THP-1 cells through the down-regulation of AKT and activation of caspase-3 [57]. Accordingly, we showed that treatment of THP-1 cells with BJe led to a significant dephosphorylation, and then inactivation, of AKT kinase, which is hyperactivated in AML [6]. Contextually, we documented that BJe increased both the p53 protein and gene expression, which could result by the action of BJe on AKT, thus suggesting the targeting of the SIRT2/AKT/p53 pathway.

Finally, in accordance with the pillars of the circular economy, the interesting pharmacological properties from re-evaluating a *Citrus* byproduct, demonstrated in this work, support the switch towards a more environmentally sustainable society.

## 5. Conclusions

Nature provides a wide range of bioactive compounds, with flavonoids standing out among the others. *Citrus* fruits are the main dietary source of flavonoids, widely cultivated, processed, and consumed throughout the world. The scenario of hematological diseases, such as AML, means we are constantly looking for innovative drugs and novel approaches, including in the landscape of natural remedies. For the first time, we showed that BJe induces anticancer effects against the human leukemia monocytic THP-1 cell line, causing cell cycle arrest in S phase and triggering the apoptotic machinery, via targeting of the SIRT2/AKT/p53 pathway. Therefore, our results encourage the study of tools in the fight against such a nefarious hematological cancer.

## Figures and Tables

**Table 1:** Oligonucleotide primer sequences used for real-time PCR.

Gene	NCBI Reference Sequence	Primer Sequence
SIRT2	NM_012237.4	Forward: 5′-TTCAAGCCAACCATCTGT-3′ Reverse: 5′-GTATCTATGTTCTGCGTGTAG-3′
TP53	NM_000546.6	Forward: 5′-GTGTGGAGTATTTGGATGAC-3′ Reverse: 5′-ATGTAGTTGTAGTGGATGGT-3′
CASP8	NM_001228.4	Forward: 5′-GTCTGTACCTTTCTGGCGGA-3′ Reverse: 5′-CTCAGGCTCTGGCAAAGTGA-3′
CASP9	NM_001229.5	Forward: 5′-GCTCAGACCAGAGATTCG-3′ Reverse: 5′-ATCCTCCAGAACCAATGTC-3′
CASP3	NM_004346.4	Forward: 5′-AGCACCTGGTTATTATTCTTGG-3′ Reverse: 5′-GCTTGTCGGCATACTGTT-3′
ACTB	NM_001101.5	Forward: 5′-TTGTTACAGGAAGTCCCTTGCC-3′ Reverse: 5′-ATGCTATCACCTCCCCTGTGTG-3′

**Table 2:** Quantitative determination of the flavonoids identified in BJe and their chemical structures.

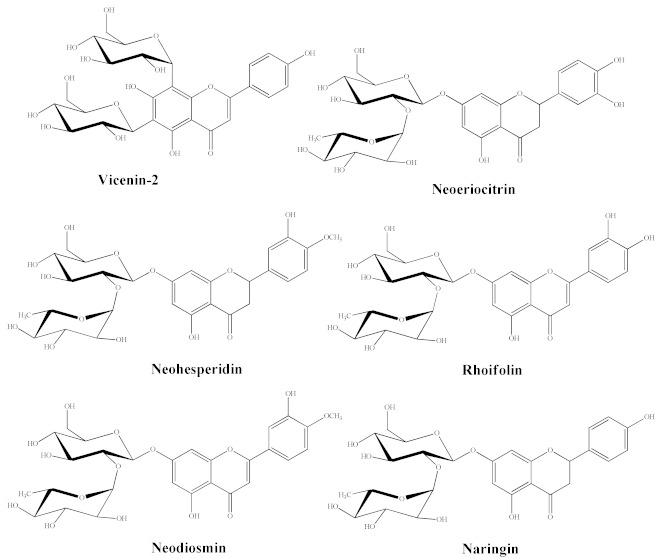			
		mg/mL (Liquid Extract)	
Peak	Compound	Mean	SD
1	Vicenin-2	<0.1	-
2	Neoeriocitrin	0.11	0.011
3	Rhoifolin	<0.1	-
4	Naringin	0.44	0.017
5	Neodiosmin	<0.1	-
6	Neohesperidin	0.59	0.037

## Data Availability

Not applicable.
